# Genetic and transformation studies reveal negative regulation of ERS1 ethylene receptor signaling in Arabidopsis

**DOI:** 10.1186/1471-2229-10-60

**Published:** 2010-04-08

**Authors:** Qian Liu, Chan Xu, Chi-Kuang Wen

**Affiliations:** 1 National Key Laboratory of Plant Molecular Genetics, Institute of Plant Physiology and Ecology, Shanghai Institutes for Biological Sciences, Chinese Academy of Sciences, Shanghai 200032, China

## Abstract

**Background:**

Ethylene receptor single mutants of Arabidopsis do not display a visibly prominent phenotype, but mutants defective in multiple ethylene receptors exhibit a constitutive ethylene response phenotype. It is inferred that ethylene responses in Arabidopsis are negatively regulated by five functionally redundant ethylene receptors. However, genetic redundancy limits further study of individual receptors and possible receptor interactions. Here, we examined the ethylene response phenotype in two quadruple receptor knockout mutants, (*ETR1*) *ers1 etr2 ein4 ers2 *and (*ERS1*) *etr1 etr2 ein4 ers2*, to unravel the functions of ETR1 and ERS1. Their functions were also reciprocally inferred from phenotypes of mutants lacking *ETR1 *or *ERS1*. Receptor protein levels are correlated with receptor gene expression. Expression levels of the remaining wild-type receptor genes were examined to estimate the receptor amount in each receptor mutant, and to evaluate if effects of *ers1 *mutations on the ethylene response phenotype were due to receptor functional compensation. As *ers1 *and *ers2 *are in the Wassilewskija (Ws) ecotype and *etr1*, *etr2*, and *ein4 *are in the Columbia (Col-0) ecotype, possible effects of ecotype mixture on ethylene responses were also investigated.

**Results:**

Ethylene responses were scored based on seedling hypocotyl measurement, seedling and rosette growth, and relative *Chitinase B *(*CHIB*) expression. Addition of *ers1 *loss-of-function mutations to any *ETR1*-containing receptor mutants alleviated ethylene growth inhibition. Growth recovery by *ers1 *mutation was reversed when the *ers1 *mutation was complemented by *ERS1p:ERS1*. The addition of the *ers2-3 *mutation to receptor mutants did not reverse the growth inhibition. Overexpressing ERS1 receptor protein in (*ETR1 ERS1*)*etr2 ein4 ers2 *substantially elevated growth inhibition and *CHIB *expression. Receptor gene expression analyses did not favor receptor functional compensation upon the loss of *ERS1*.

**Conclusions:**

Our results suggest that ERS1 has dual functions in the regulation of ethylene responses. In addition to repressing ethylene responses, ERS1 also promotes ethylene responses in an ETR1-dependent manner. Several lines of evidence support the argument that ecotype mixture does not reverse ethylene responses. Loss of *ERS1 *did not lead to an increase in total receptor gene expression, and functional compensation was not observed. The inhibitory effects of ERS1 on the ethylene signaling pathway imply negative receptor collaboration.

## Background

Ethylene plays important roles in many aspects of plant growth and development, including fruit ripening, senescence and pathogen responses, and nodulation in *Medicago *[1-6]. Ethylene induces the expression of *Sub1A *or *SNORKEL1*/*SNORKEL2 *in certain rice cultivars, allowing them to survive flooding by various mechanisms [7,8]. Arabidopsis has been used as a model plant for the study of ethylene signal transduction for the past two decades. Air-grown, etiolated Arabidopsis seedlings have a long seedling hypocotyl and primary root. In the presence of ethylene, seedling growth is substantially inhibited, and the hypocotyl and primary root become shorter. In addition, ethylene treatment induces the apical hook formation that is caused by exaggerated curvature at the apical region [9]. In the adult stage, ethylene treatment inhibits rosette leaf growth. Mutants defective in multiple ethylene receptors or in Constitutive Triple-Response1 (CTR1), a mitogen-activated protein kinase kinase kinase (MAPKKK) protein acting directly downstream of the receptors, display a constitutive ethylene response phenotype with substantially inhibited rosette leaf growth [10]. When grown under light without exogenous sucrose, mutants displaying a constitutive ethylene response phenotype have small and compact cotyledons and the seedling hypocotyl and primary root are shorter than wild type [11]. The hypocotyl length of etiolated seedlings, growth of light-grown seedlings, and adult rosette phenotype can be used to score for ethylene responses in Arabidopsis [12-16].

Arabidopsis has five ethylene receptors, which are structurally similar to the His-kinase proteins that are prevalently found in two-component modules in prokaryotes and some lower eukaryotes [12,17-19]. The five ethylene receptors are structurally distinct and can be classified into two subfamilies. ETR1 and ERS1 are in subfamily I, and ETR2, EIN4, and ERS2 are in subfamily II. Subfamily I receptors have three putative transmembrane domains and a His-kinase domain, which has the signature motifs essential to His-kinase activity. Subfamily II receptors have three or four putative transmembrane domains, depending on the algorithms used for topological prediction, and a non-conserved His-kinase domain, in which some consensus amino acid residues essential for His-kinase activity are lacking [11,20].

Biochemical studies indicate that ETR1 has His-kinase activity and all subfamily II receptors have Ser/Thr kinase activity. ERS1 has both His-kinase and Ser/Thr kinase activities. ERS1 is believed to possess Ser/Thr kinase activity because histidine autophosphorylation is lost when ERS1 is assayed in the presence of both Mg^2+^and Mn^2+ ^[20]. Mutants lacking both *ETR1 *and *ERS1 *display extremely strong ethylene growth inhibition, implying unique roles of subfamily I members in the ethylene signaling [11,16,21]. Expression of the kinase-dead etr1 [HGG] isoform is able to reverse the *etr1-7 ers1-2 *growth inhibition, indicating that His-kinase activity is not essential to ETR1 receptor signaling [16]. In addition, expression of the truncated etr1(1-349) fragment substantially reverses the *etr1-7 ers1-2 *mutant phenotype, suggesting that wild-type ETR1 receptor signaling can be mediated through the N terminus, possibly via the GAF domain [11].

Some ethylene receptors can be regulated at transcriptional and/or translational levels in Arabidopsis. Expression of TAP-tagged receptors suggests a correlation of receptor protein amount and transcript level in air-grown Arabidopsis [22]. Ethylene treatment or receptor gene mutations do not alter ETR1 protein or *ETR1 *transcript levels [23]. Expression of *ERS1, ERS2 *and *ETR2 *can be induced by ethylene treatment [17,22]. ETR2 protein accumulates at a high ethylene concentration (10 μL L^-1^) and undergoes protein degradation when ethylene concentration exceeds 100 μL L^-1 ^[22]. The ethylene-binding test, at a very low ethylene concentration (0.1 μL^-1^), suggests that receptor amount is relevant to receptor gene expression level, and that up-regulation of remaining receptors in a receptor mutant does not functionally compensate for the defective ones [24]. The *etr1-7 ers1-2 *mutant displays extremely strong growth inhibition; ectopic expression of any of the subfamily II receptors cannot reverse the mutant phenotype [16]. Together, these studies suggest that ethylene receptors may act synergistically and are not functionally replaceable by other receptors.

As single ethylene mutants do not display a visibly prominent phenotype, ethylene receptor function is inferred from phenotypes of mutants defective in multiple receptors [12]. To infer the functions of the single receptor genes *ETR1 *and *ERS1*, we characterized the ethylene response phenotype in (*ETR1*)*ers1 etr2 ein4 ers2 *and (*ERS1*)*etr1 etr2 ein4 ers2*. Effects of the respective loss of *ETR1 *and *ERS1 *on ethylene response phenotypes were also reciprocally examined. We found that ERS1 can promote ethylene responses in the presence of ETR1. The possibility that ecotype mixture might alleviate growth inhibition was not favored. Because ERS1 is also able to repress ethylene responses [11,12], we hypothesized that ERS1 has dual roles in the regulation of ethylene signaling.

## Results

### Ethylene response phenotype in ethylene receptor mutants

Dark-grown wild-type seedlings have a long hypocotyl when germinated in air. Ethylene treatment inhibits the hypocotyl elongation, and mutants defective in multiple ethylene receptor genes display various degrees of hypocotyl growth inhibition [12]. In this study, seedling hypocotyl length was measured to evaluate effects of the respective loss of *ETR1 *and *ERS1 *on the ethylene response. If not specified, *ers1 *refers to the *ers1-2 *allele, and loss-of-function receptor mutants were studied throughout this work.

**Figure 1:**
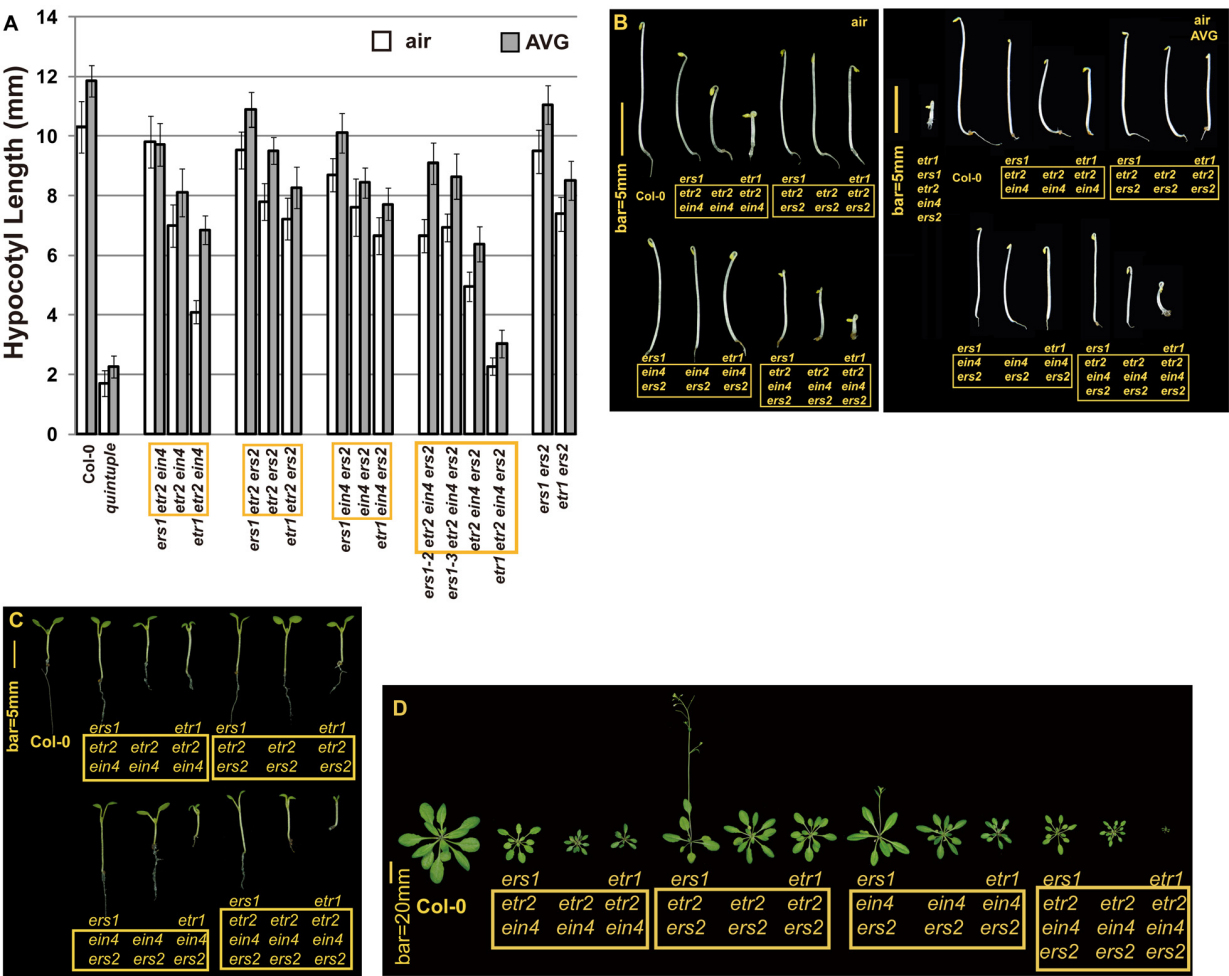
**Seedling hypocotyl measurement and phenotypes of ethylene receptor mutants**. (A) seedling hypocotyl length of air-grown seedlings in the presence and absence of the ethylene biosynthesis inhibitor AVG. (B) etiolated and (C) light-grown seedlings, and (D) rosettes of ethylene receptor mutants. Common receptor mutations are highlighted in the yellow box for each set of mutants. Error bars indicate standard deviation. Quintuple: the quintuple receptor mutant.

In all receptor mutant sets examined, the addition of the *etr1-7 *null mutation resulted in hypocotyl growth inhibition, while the addition of the *ers1-2 *loss-of-function mutation resulted in hypocotyl growth recovery (Figures 1A and 1B; Table 1 for LSD-t test). In addition to the dark-grown triple-response phenotype, the phenotype of light-grown seedlings was examined. The wild-type seedlings had a long primary root and fully extended cotyledons when grown under light. Mutants lacking multiple receptors displayed various degrees of growth inhibition. In several receptor knockout combinations, the addition of *ers1-2 *resulted in growth recovery while the addition of *etr1-7 *caused growth inhibition. Among those mutants, (*ERS1*)*etr1 etr2 ein4 ers2 *displayed the strongest growth inhibition; in contrast, growth inhibition was minor in (*ETR1*) *ers1 etr2 ein4 ers2 *(Figure 1C).

**Table 1:** LSD-t test for seedling hypocotyl measurements shown in Figure 1A. Seedlings were grown in air or in the presence of AVG

Paired Comparison (air)		*LSD-t*	*df*	*P*
*etr2 ein4*	*ers1 etr2 ein4*	64.25	84	3.41 × 10^-73^
*etr2 ein4*	*etr1 etr2 ein4*	85.22	114	2.35 × 10^-86^
*etr2 ers2*	*ers1 etr2 ers2*	24.85	81	1.19 × 10^-39^
*etr2 ers2*	*etr1 etr2 ers2*	8.24	111	2.41 × 10^-12^
*ein4 ers2*	*ers1 ein4 ers2*	13.61	88	2.34 × 10^-23^
*ein4 ers2*	*etr1 ein4 ers2*	11.96	119	3.23 × 10^-20^
*etr2 ein4 ers2*	*ers1-3 etr2 ein4 ers2*	38.62	132	7.67 × 10^-55^
*etr2 ein4 ers2*	*ers1-2 etr2 ein4 ers2*	42.17	83	7.52 × 10^-79^
*etr2 ein4 ers2*	*etr1 etr2 ein4 ers2*	53.78	113	4.20 × 10^-69^

**Paired Comparison (AVG)**		***LSD-t***	***df***	***P***

*etr2 ein4*	*ers1 etr2 ein4*	18.38	58	7.28 × 10^-26^
*etr2 ein4*	*etr1 etr2 ein4*	14.54	58	5.36 × 10^-21^
*etr2 ers2*	*ers1 etr2 ers2*	18.24	58	1.08 × 10^-25^
*etr2 ers2*	*etr1 etr2 ers2*	16.38	58	2.00 × 10^-23^
*ein4 ers2*	*ers1 ein4 ers2*	24.63	91	2.80 × 10^-42^
*ein4 ers2*	*etr1 ein4 ers2*	8.38	57	1.61 × 10^-11^
*etr2 ein4 ers2*	*ers1-3 etr2 ein4 ers2*	29.54	74	1.25 × 10^-48^
*etr2 ein4 ers2*	*ers1-2 etr2 ein4 ers2*	35.83	71	1.69 × 10^-41^
*etr2 ein4 ers2*	*etr1 etr2 ein4 ers2*	38.61	56	4.75 × 10^-42^

*LSD-t*: the *t *value for a paired comparison in a set of ethylene receptor knockout mutants.

*df*: degree of freedom. *P*: probability of a numerically larger value of *t*.

air: seedlings germinated in air; AVG: seedlings germinated in the presence of AVG.

Our results implied that ERS1 has positive effects on ethylene responses, in contrast to previous studies showing that ERS1 can inhibit ethylene responses [11,16,21]. However, endogenous ethylene production may affect the analyses. AVG (L-α-(2-aminoethoxyvinyl)glycine) is an effective ethylene biosynthesis inhibitor and reduces ethylene production from 6.74 ± 0.1 nL (untreated) to 0.41 ± 0.60 nL (AVG-treated) in etiolated wild-type seedlings [9]. In this study, AVG was included to eliminate endogenous ethylene production, and the effects of ERS1 on the seedling triple response were evaluated. Consistent with our results, in any *ETR1*-containing receptor mutants, the loss of *ERS1 *substantially led to hypocotyl elongation. In contrast, the *etr1-7 *null mutation caused growth inhibition in corresponding *ERS1*-containing mutant sets. (*ERS1*)*etr1 etr2 ein4 ers2 *displayed a dramatic inhibition of hypocotyl elongation. The growth inhibition in *etr2 ein4 ers2 *was greatly relieved upon the addition of the hypomorphic *ers1-2 *or the amorphic *ers1-3 *allele (Figures 1A and 1B; Table 1 for LSD-t test).

The loss of multiple ethylene receptors leads to dramatic reduction in rosette size, due to the loss of repression in ethylene response by those receptors [11,13,16,24-26]. The rosette phenotype of receptor-deficient mutants was examined to score for ethylene responses. Mutants that exhibited inhibition in rosette growth upon the loss of *ETR1 *consistently displayed growth recovery upon the loss of *ERS1*. Among those mutants, (*ERS1*) *etr1 etr2 ein4 ers2 *displayed the strongest growth inhibition, and (*ETR1*) *ers1 etr2 ein4 ers2 *had a larger rosette than *etr2 ein4 ers2*. *ers1 etr2 ers2 *exhibited early flowering and rosette growth ceased (Figure 1D). Both the *ers1 *and *ers2 *mutations are from the Wassilewskija (Ws) ecotype, and the other receptor mutations are from the Columbia (Col-0) ecotype [12,16,17,21]. The early-flowering phenotype could be a trait from Ws background, because Ws exhibited early bolting in our study (data not shown). Effects of the *ers1 *mutation on *etr2 ers2 *growth were not determined in this study.

Our data show that the addition of the *ers1 *loss-of-function mutation to any *ETR1*-containing receptor mutants alleviates growth inhibition to various degrees. The extent of growth inhibition in mutants lacking ETR1 or ERS1 varied, supporting the hypothesis that the receptors function synergistically [24].

### Receptor gene expression in receptor mutants

Studies on ethylene binding (at a relatively low concentration of ethylene) as well as the expression of TAP-tagged receptors (in air) indicate that receptor protein levels are correlated with receptor gene expression [22,24]. Our genetic analyses indicated that the respective loss of *ETR1 *and *ERS1 *has an opposite effect on the ethylene response phenotype. To investigate if the loss of *ERS1 *results in an increase in total receptor gene expression, and thus alleviates growth inhibition, we estimated the total receptor amount in the receptor mutants by measuring the expression of remaining wild-type receptor genes.

**Figure 2:**
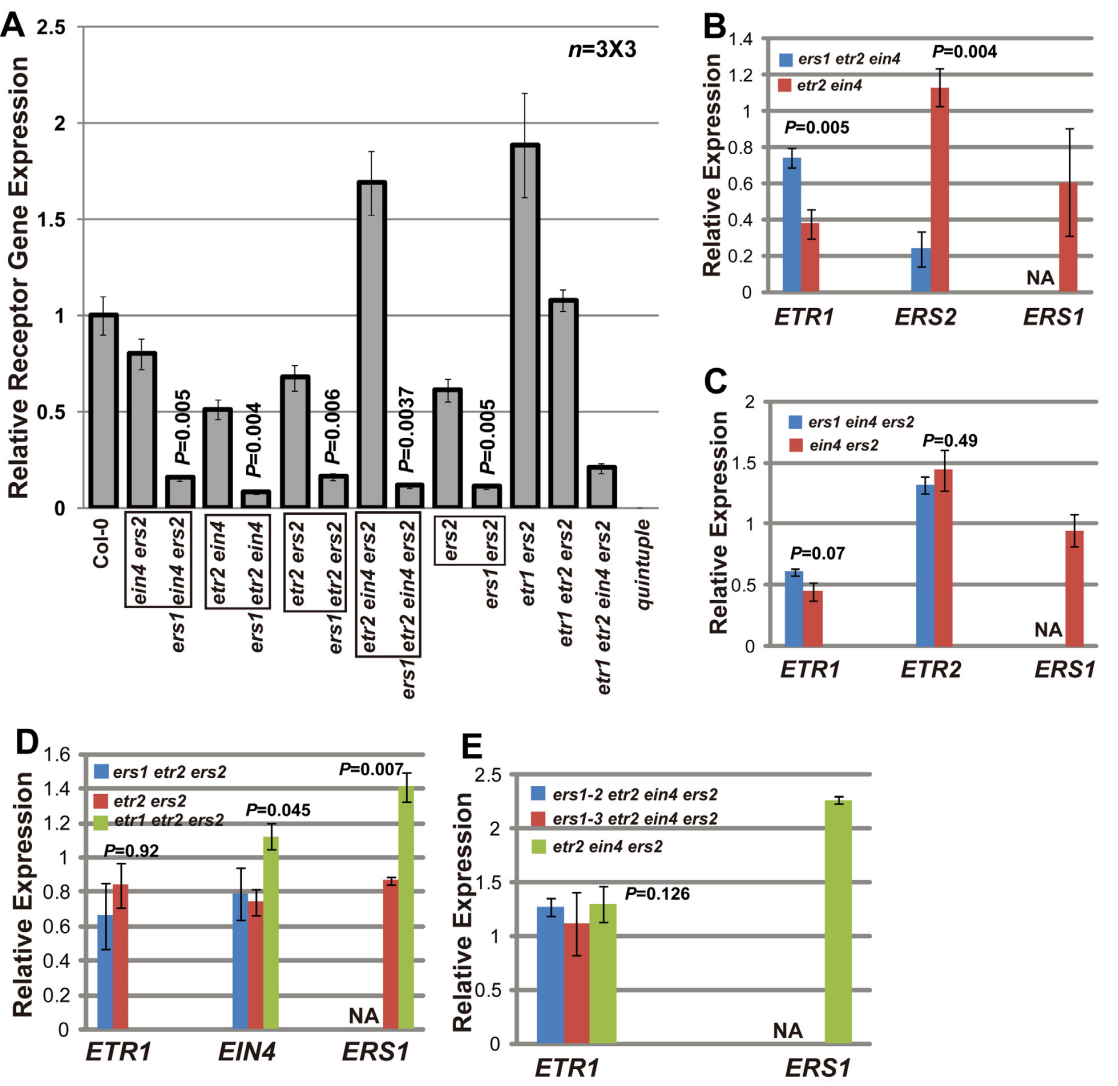
**Relative receptor gene expression in wild type and ethylene receptor mutants**. (A) expression of remaining wild-type receptor genes in receptor mutants relative to total receptor gene expression in wild type (Col-0). *P *value indicates the probability of a numerically larger value of *t *for the comparison (Student's *t *test) between two isogenic mutants highlighted with a box. (B), (C), (D), and (E) expression of individual receptor genes in isogenic receptor mutants relative to that in wild type. Box highlights the common receptor gene mutations of a set of isogenic mutants. Error bars indicate standard deviation. *n *= 3 × 3: each measurement was repeated three times from three independent biological materials. NA: the *ERS1 *expression is not measured in *ers1 *mutants. *P*: probability of a numerically larger value of *t *in a LSD-t test.

Gene expression was measured by real-time fluorescence quantitative RT-PCR (qRT-PCR). Figure 2A shows that in any receptor mutant sets, the loss of *ERS1 *resulted in a reduction in total receptor gene expression (Student's *t *test for each isogenic mutant pair and *P *< 0.006) in adult rosette leaves. Notably, *etr2 ein4 *and *etr2 ein4 ers2 *had a smaller rosette size and higher total receptor gene expression than *ers1 etr2 ein4 *and *ers1 etr2 ein4 ers2*, respectively (Figures 1D and 2A). The *etr2 ein4 ers2 *mutant displayed a constitutive ethylene response phenotype and its relative receptor gene expression was greater than that in wild type (Col-0; Student's *t *test and *P *< 0.01). The total receptor gene expression in (*ERS1*)*etr1 etr2 ein4 ers2 *was statistically identical to that in *ers1 ein4 ers2*, *ers1 etr2 ein4*, *ers1 etr2 ein4*, *ers1 ers2*, and *ers1 etr2 ein4 ers2 *(LSD-t test and *P *> 0.49 for each paired comparison); however, it had the smallest rosette size (Figures 1D and 2A).

The relative expression of each remaining receptor gene upon the loss of *ERS1 *was analyzed by assigning the corresponding gene expression in the wild-type (Col-0) rosette the value of 1. In *etr2 ein4*, the relative *ERS2 *and *ETR1 *expression levels were 1.13 and 0.38, respectively, and changed to 0.24 and 0.74 upon the addition of the *ers1-2 *mutation (Figure 2B). The changes did not result in elevation of total receptor gene expression (Figure 2A). In addition, *ERS2 *expression is ethylene-inducible [17] and our data show that *etr2 ein4 *had a higher *ERS2 *level than *ers1 etr2 ein4*. These results imply that the ethylene response was stronger in *etr2 ein4 *than in *ers1 etr2 ein4*, in agreement with the ethylene growth inhibition phenotype (Figure 1). For the other three mutant sets, the loss of *ERS1 *did not result in significant alterations in the expression of the remaining receptor genes (Figures 2C, 2D, and 2E). Notably, *ETR1 *expression in *etr2 ein4 ers2 *was identical to that in *ers1-2 etr2 ein4 ers2 *and *ers1-3 etr2 ein4 ers2 *(*F *test and *P *= 0.126); the latter two mutants had a larger rosette. The *etr1 etr2 ers2 *mutant had the same total receptor gene expression levels as Col-0 (Figure 2A; Student's *t *test and *P *= 0.144) due to elevation in *ERS1 *and *EIN4 *expression levels (Figure 2B).

Our results show that the loss of *ERS1 *does not result in transcriptional compensation by other receptor genes, and that higher receptor gene expression levels do not necessarily lead to a greater extent of growth recovery. The elevated *ERS1 *and *EIN4 *expression did not functionally compensate for the *etr1*, *etr2*, and *ers2 *mutations. Receptor mutants carrying *ERS1 *had a higher receptor gene expression than those carrying *ers1*, largely excluding the possibility of functional compensation at transcriptional or translational levels. The expression of those knockout genes was not measured in this study; the possibility that they may have residual function is very slim due to the nature of their mutations.

### Effects of *ers1 *alleles on growth recovery in receptor mutants

The above data show that the *ers1-2 *loss-of-function mutation caused growth recovery in receptor mutants. However, the *ers1-2 *mutation is hypomorphic while *ers1-3 *is amorphic [11,21]; therefore, the allele specificity needed to be examined.

**Figure 3:**
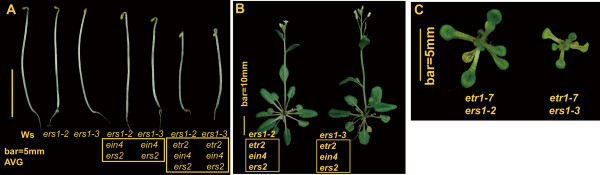
**Effects of *ers1 *alleles on ethylene receptor mutant growth**. (A) phenotype of dark-grown seedlings in the presence of AVG. (B), (C) adult phenotype of *ers1-2 *and *ers1-3 *mutants. Box highlights common mutations.

To examine if *ers1-2 *and *ers1-3 *could result in any significant difference in growth recovery, the mutant phenotypes of *ers1-2 *and *ers1-3 *were compared throughout developmental stages. They were phenotypically indiscernible (Figures 1A, 3A and 3B), except that the *etr1 ers1-2 *double mutant displayed a milder mutant phenotype than *etr1 ers1-3 *(Figure 3C), as shown in previous studies [11,21]. The hypocotyl lengths of etiolated *ers1-2 etr2 ein4 ers2 *and *ers1-3 etr2 ein4 ers2 *were nearly identical when germinated in air or in the presence of AVG (Student's *t *test and *P *= 0.01). Because the addition of *ers1 *to *etr1-7 *did not reverse the ethylene growth inhibition (Figure 3C), and growth recovery by *ers1 *only occurred in the presence of *ETR1*, the effects on the alleviation of growth inhibition by *ers1 *are *ETR1 *dependent. Notably, the addition of *ers1 *to *etr1 etr2 ein4 ers2 *also led to growth inhibition in the quintuple receptor knock mutant regardless of AVG treatment (Figure 1A; Student's *t *test and P < 10^-9^).

### Effects of ecotype mixture on growth recovery by *ers1 *mutations

The *ers1-2 *and *ers1-3 *mutations are in the Ws ecotype, while *etr1*, *etr2*, and *ein4 *are in the Col-0 ecotype. In Arabidopsis, heterosis may result in an increase of biomass [27]; thus, we needed to exclude the possibility that a mix of the two ecotypes could alter the ethylene response phenotype.

**Figure 4:**
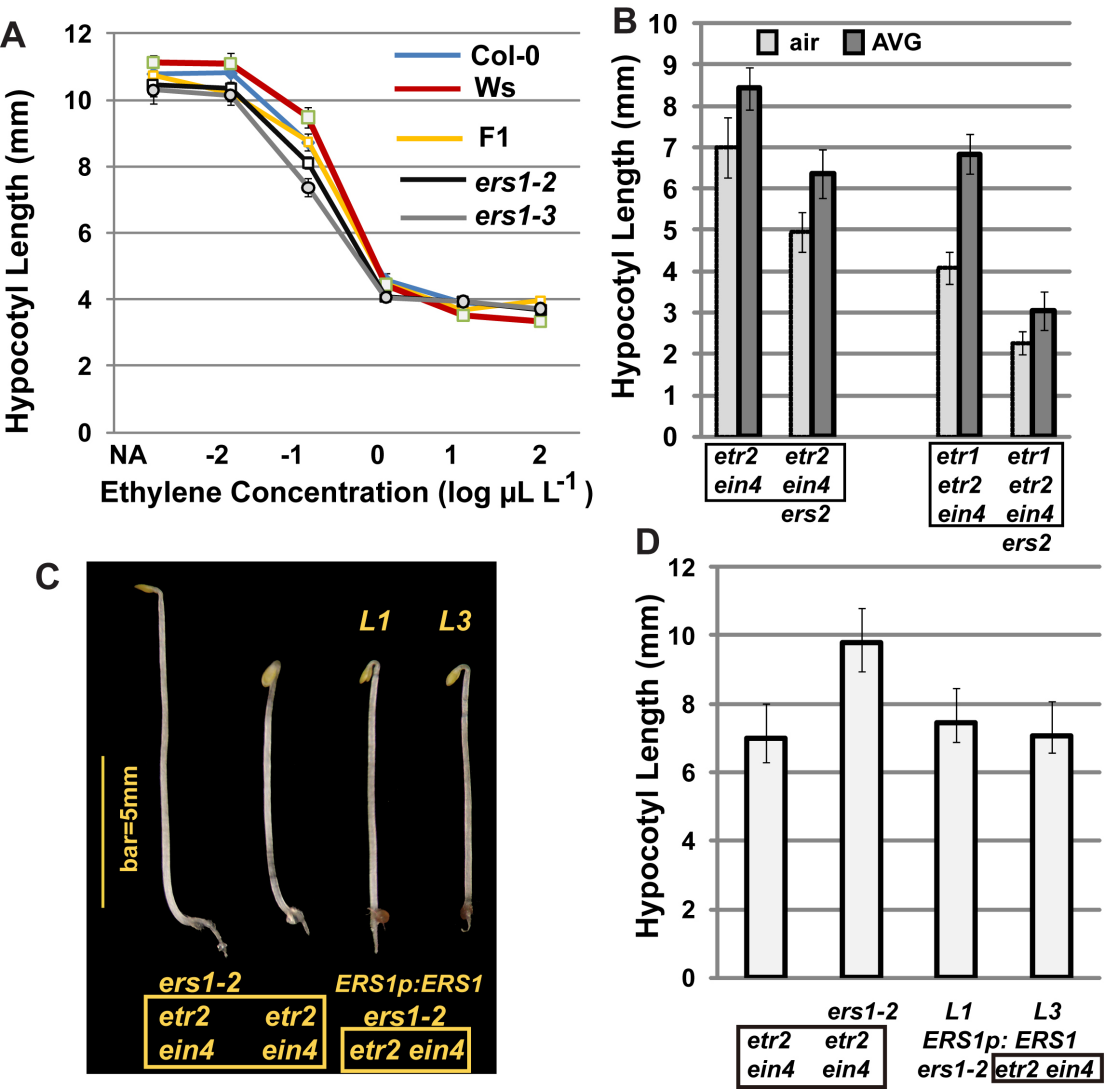
**Effects of ecotype mixture on *ers1*-mediated growth recovery**. (A) ethylene dose-response assay for *ers1-2*, *ers1-3*, Col-0, Ws, and the F1 of Col-0 and Ws. (B) seedling hypocotyl measurement of mutants respectively carrying and lacking *ERS2*. (C) phenotype and (D) hypocotyl measurement of etiolated seedlings of *ers1-2 etr2 ein4*, *ERS1p:ERS1 ers1-2 etr2 ein4*, and *etr2 ein4*. Error bars indicate standard deviation. *L*1 and *L*3: two independent *ERS1 *transformation lines. Box indicates common mutations.

A dose-response curve was drawn to compare ethylene response in Col-0, Ws, and their F1 (first filial). These lines exhibited a nearly identical dose-response curve over a wide range of ethylene concentrations (6 logs); the seedling hypocotyl lengths of Col-0 and the F1 were statistically identical at the ethylene concentration range of 0-1 μL L^-1 ^(LSD-t test and *P *= 0.04 to 0.75), except that at 10^-2 ^μL L^-1 ^ethylene the F1 seedling was much shorter. Additionally, the Col-0 seedling was only 0.2 mm longer than the F1 at 10 and 100 μL L^-1 ^ethylene (LSD-t test and *P *< 10^-6^; Figure 4A). These results tell us that the mixture of Col-0 and Ws is not sufficient to significantly reverse ethylene growth inhibition.

ERS1 is shown to repress ethylene responses [11,16,21]; in our study, the loss of *ERS1 *reversed ethylene growth inhibition. We examined how the loss of *ERS1 *may affect ethylene growth inhibition in wild type (Ws). At a low ethylene concentration range (below 1 μL L^-1^), both *ers1-2 *and *ers1-3 *had a shorter seedling hypocotyl than Ws, and the difference was the greatest (1.4 and 2.1 mm; LSD-t test and *P *< 10^-27^) at 0.1 μL L^-1 ^ethylene. At a high ethylene concentration range (10-100 μL L^-1^), ethylene growth inhibition in *ers1-2 *and *ers1-3 *was weaker than in Ws (Figure 4A; LSD-t test and *P *< 10^-11^). *ers1-3 *and *ers1-2 *had identical seedling hypocotyl lengths over a wide range of ethylene concentrations (6 logs; LSD-t test and *P *= 0.1 to 0.9), except that *ers1-3 *was shorter (0.7 mm; LSD-t test and *P *< 10^-14^) than *ers1-2 *at 0.1 μL L^-1 ^ethylene.

In addition to *ers1 *mutations, *ers2 *is also in the Ws ecotype. Mutants lacking *ERS2 *had a shorter seedling hypocotyl than those carrying *ERS2*, providing evidence that growth recovery in *ERS1*-lacking mutants is not due to ecotype mixture (Figure 4B; Student's *t *test and *P *< 0.01).

Possible effects of ecotype mixture on the ethylene response phenotype were reciprocally examined by complementing the *ers1-2 *mutation with *ERS1*. The *ERS1p:ERS1 *transgene was cloned from Col-0. Two independent *ERS1p:ERS1 ers1-2 etr2 ein4 *transformation lines phenotypically resembled *etr2 ein4*, and exhibited a shorter hypocotyl than *ers1-2 etr2 ein4 *(Figures 4C and 4D; LSD-t test and *P *< 10^-27^).

Our results suggest that the repression of growth inhibition upon the loss of *ERS1 *is not due to the mix of Col-0 and Ws backgrounds. The ethylene dose-response assay for *ers1 *and Ws implies that ERS1 may repress ethylene growth inhibition at low ethylene concentrations, but promote ethylene responses at high ethylene concentrations in wild type.

### ERS1 overexpression exaggerates growth inhibition in *etr2 ein4 ers2*

ERS1 is essential for repression of the ethylene response in the *etr1-7 *loss-of-function mutant (Figure 3C) [11,16,21]. Our genetic analysis shows that ERS1 can elevate ethylene response in receptor mutants carrying ETR1, indicating that ERS1 has dual functions in the regulation of the ethylene response. Conceivably, ERS1 overexpression elevates growth inhibition in *etr2 ein4 ers2*, regardless of the ecotype or ecotype mixture.

To test our hypothesis, *ERS1 *was expressed under the constitutive cauliflower mosaic virus (*CaMV*) *35S *promoter in (*ETR1 ERS1*)*etr2 ein4 ers2*. Two classes of phenotype (A and B) were typical among the resulting transformed lines. Class A phenotypically resembled *etr2 ein4 ers2*, while class B plants were sterile and the rosette was much smaller. Efforts were made to express *ERS1 *under an inducible promoter to obtain stable expression lines; however, the resulting siblings of any homozygous transformation lines exhibited various degrees of elevation in growth inhibition, possibly due to unstable expression (data not shown). We thus examined *35S:ERS1 *transformation lines.

**Figure 5:**
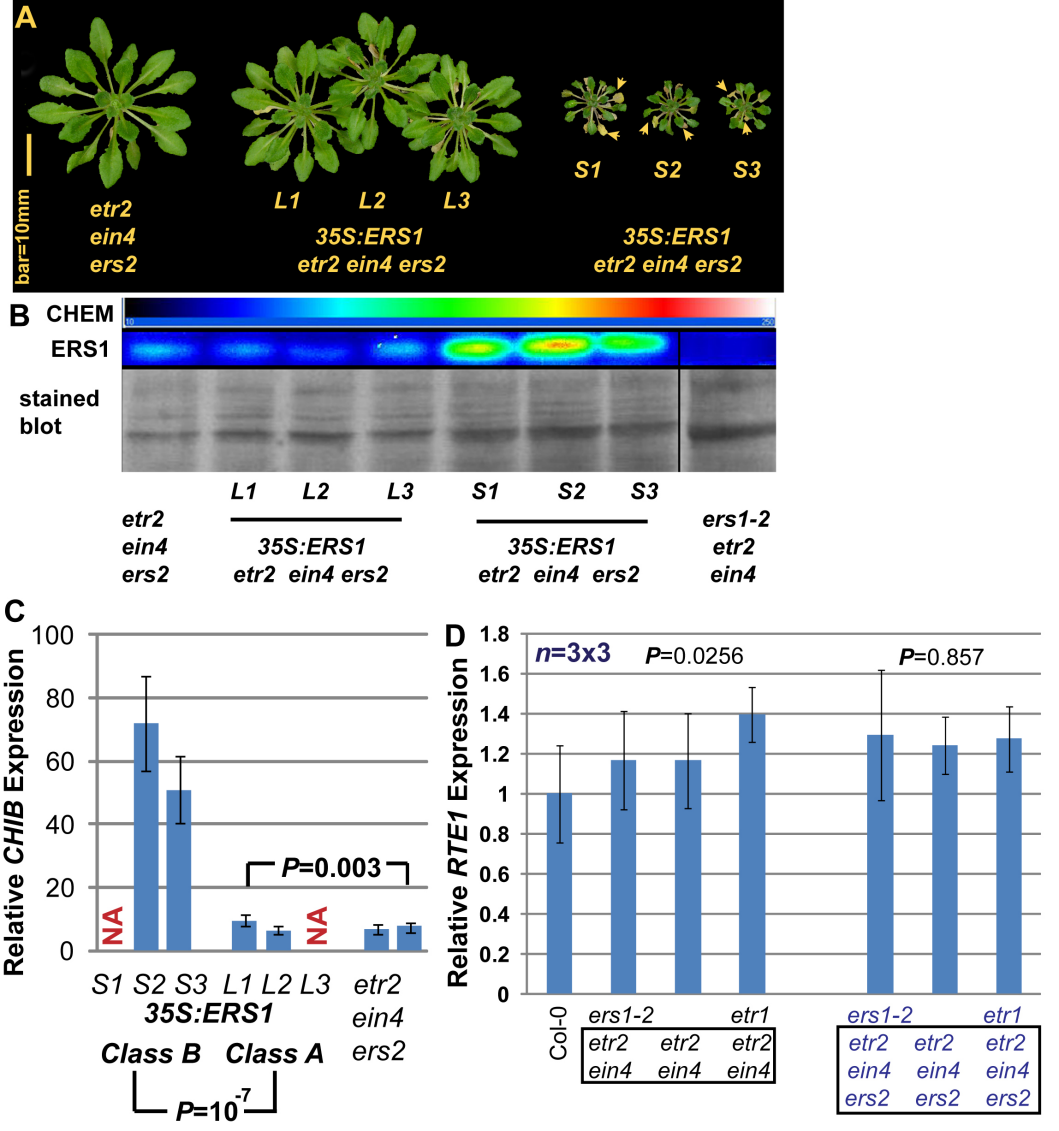
**Effect of ERS1 overexpression on *etr2 ein4 ers2***. (A) adult phenotype of *etr2 ein4 ers2*, class A (*L*1, *L*2, and *L*3) and class B (*S*1, *S*2, and *S*3) lines carrying the *35S:ERS1 *transgene. (B) immunoassay of ERS1 level in individual transformed lines. The ERS1 protein is not detectable in the *ers1 *mutant *ers1-2 etr2 ein4*. (C) relative *CHIB *gene expression in *etr2 ein4 ers2 *and transformed lines. (D) relative *RTE1 *gene expression in receptor mutants. Chemiluminance for the immunoassay was captured by a cold CCD (at -110°C and displayed in pseudo-color; the pseudo-color bar (CHEM) indicates relative signal strength from weak (dark) to strong (bright). ERS1: relative ERS1 accumulation in immunoassay probed with ERS1 antibody. Stained blot: membrane was stained with Coomassie Blue after the immunoassay to indicate relative protein amount. NA: data not available. Arrows indicate senesced leaves. Error bars indicate standard deviation. *P*: probability of a larger *F *value. *n *= 3 × 3: each measurement was repeated three times from three independent biological materials. Common mutations are highlighted in a box.

Due to infertility, three independent class B lines in the T1 generation were characterized. In addition to the extremely small rosette phenotype, in comparison to that of *etr2 ein4 ers2*, class B lines displayed early leaf senescence (Figure 5A), a phenotype of the ethylene response [12,13,15,28]. The relative expression level of ERS1 protein to the rosette phenotype was examined. Class A lines exhibited a similar ERS1 protein level as *etr2 ein4 ers2*, while class B lines had much higher ERS1 protein accumulation (Figure 5B). Expression of ethylene-inducible *CHIB *[12] was estimated by qRT-PCR to examine the relationship between ethylene response and rosette phenotype of class B lines. In comparison to wild type, *CHIB *expression in *etr2 ein4 ers2 *and the class A lines was elevated to 6-fold, while expression in Class B lines was 50- to 70-fold (Figure 5C). These results support our genetic analyses and the hypothesis that ERS1 can negate the repression of the ethylene response by ETR1. In addition, these results back the argument that the growth inhibition caused by ERS1 is not due to ecotype mixture.

We previously showed that RTE1 is able to elevate ETR1 signaling [13]. We next investigated whether ERS1 could modulate *RTE1 *expression to promote the ethylene response repressed by ETR1. When estimated by qRT-PCR, the *ers1-2 *mutation did not significantly increase *RTE1 *expression relative to the corresponding *ERS1*-containing mutants (Figure 5D). *etr1 etr2 ein4 *exhibited slightly higher *RTE1 *expression than *ers1 etr2 ein4 *and *etr2 ein4*; two quadruple receptor mutants had similar *RTE1 *expression.

### Seedling hypocotyl growth recovery by *ers1 *is not inhibited by ethylene

Ethylene binding is known to inactivate ethylene receptors [29]. We investigated whether ethylene binding negates the inhibitory effect of ERS1 on plant growth.

**Figure 6:**
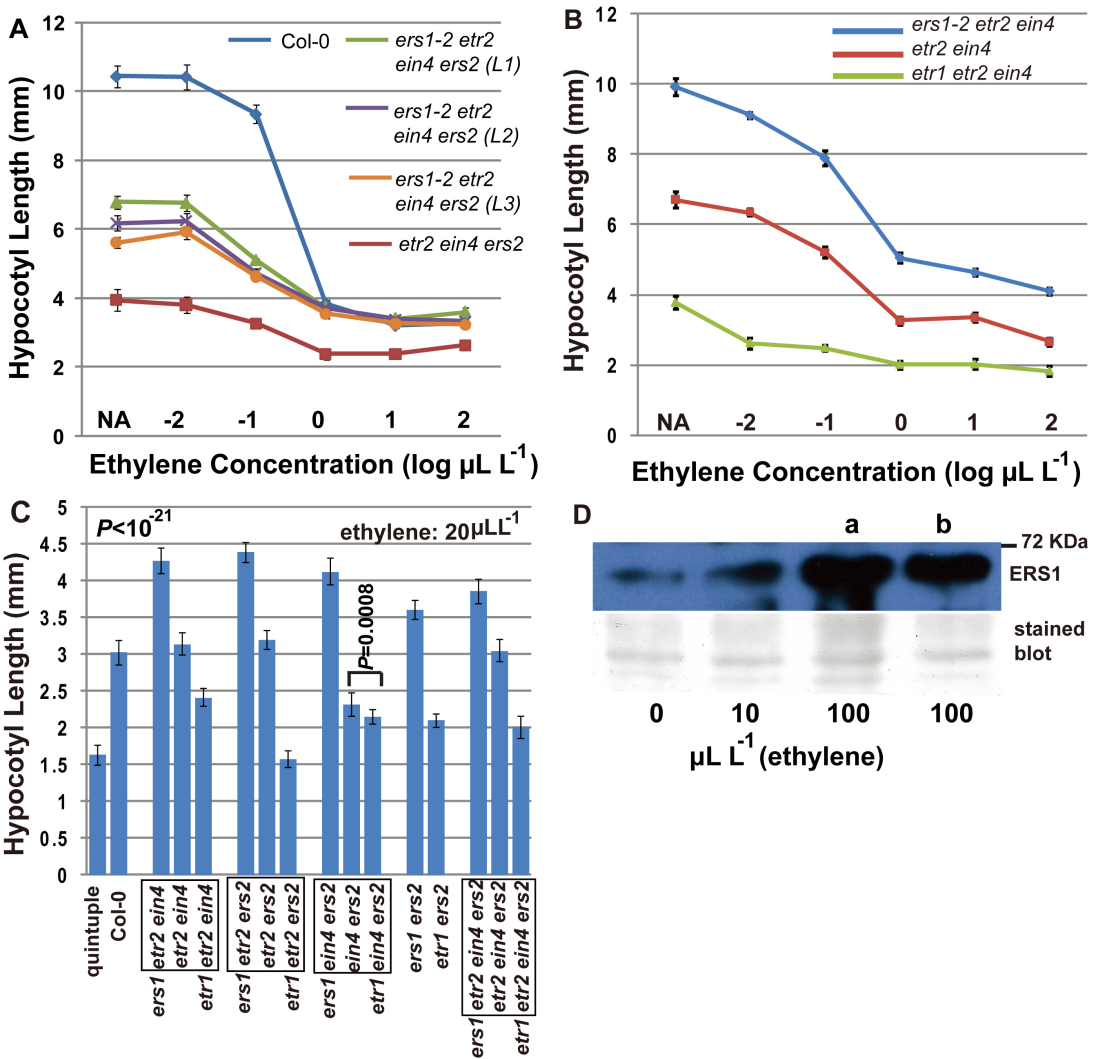
**Inhibitory effect of ERS1 on the repression of the ethylene response is not blocked by ethylene**. (A), (B) ethylene dose-response curve for two sets of *ERS1*-lacking mutants. (C) under a high ethylene concentration, ERS1 still exerts an inhibitory effect on seedling hypocotyl elongation. (D) ERS1 accumulation is elevated upon ethylene treatment; a and b represent ERS1 level from two independent wild-type plants. Error bar indicates standard deviation. ERS1: the ERS1 protein. NA: no ethylene treatment. Stained blot: membrane was stained with Coomassie Blue after the immunoassay to indicate relative protein amount. *L*1, *L*2, and *L*3 represent three independently identified isogenic mutants. A molecular weight marker is indicated at the position of 72 kD. Common mutations are highlighted in a box. *P*: probability of a numerically larger *t *in Student's *t *test or LSD-t test.

In an ethylene dose-response assay, three independently obtained isogenic receptor mutant lines lacking *ERS1 *had a longer seedling hypocotyl than the corresponding mutants containing *ERS1 *(Figure 6A; LSD-t test and *P *< 10^-19^). Minor variations in the hypocotyl lengths among the three individual lines at low ethylene ranges (0-0.1 μL L^-1^; *F *test and *P *< 10^-8^) did not affect the conclusion that these *ers1*-lacking mutants had a much longer seedling hypocotyl than the *ERS1*-containing plants. We do not exclude the possibility that the variations could be due to ecotype mixture. *ers1-2 etr2 ein4 *was consistently longer than *etr2 ein4 *over the same ethylene concentration range, while *etr1 etr2 ein4 *was the shortest (Figure 6B; LSD-t test and *P *< 10^-21^). At a high ethylene concentration (20 μL L^-1^), *ERS1*-lacking mutants always had a longer seedling hypocotyl than corresponding *ERS1*-containing mutants. Notably, the corresponding *ETR1*-lacking mutants had the shortest seedling hypocotyl in any receptor mutant sets (Figure 6C; Student's *t *test or LSD-t test and *P *< 0.01).

Ethylene has various effects on the steady-state receptor amount. The ETR1 receptor amount is unaltered in an array of receptor mutants [23] or by ethylene treatment [30]. *EIN4 *expression is not ethylene inducible [17]. The ETR2 receptor amount decreases at high ethylene concentrations in hydroponically grown Arabidopsis [30]. We examined the ERS1 receptor amount by immunoanalysis and Figure 6D shows that ERS1 accumulation was coupled with an increase in ethylene concentration. These results suggest that the steady-state ERS1 receptor did not undergo bulk degradation upon ethylene binding. Additionally, elevation in ERS1 protein levels under ethylene treatment may contribute to growth inhibition, explaining why *ers1 *mutants exhibited weaker growth inhibition than Ws at high ethylene concentrations (Figure 4A).

Our results show that the effect of the loss of *ERS1 *on growth recovery was similar in all five sets of receptor mutants at high ethylene concentrations. Ligand binding did not induce bulk ERS1 degradation, nor block the growth inhibition by ERS1. In addition, ethylene-treated receptor mutants exhibited different seedling hypocotyl lengths, suggesting that the ethylene receptors are not equally inactivated by ethylene. Alternatively, the signaling ability of each receptor member may differ.

## Discussion

Previous genetic analyses implied that ethylene responses are negatively regulated in Arabidopsis by five ethylene receptors, which are functionally redundant and not exchangeable [11,12,16,21]. Unexpectedly, our results suggest that ERS1 can also promote ethylene responses. The underlying mechanism for the positive effect of ERS1 on ethylene responses needs further investigation. Currently, the nature of the ethylene receptor signal is unknown, limiting further biochemical studies on the mechanisms of negative regulation by ERS1. ETR1 and ERS1 can form a heteromeric complex [22]; our data and other studies suggest that the unique ERS1 function is ETR1 dependent, because the addition of *ers1 *to the *etr1-7 *null mutant does not result in growth recovery. One explanation is that ERS1 may partially negate ETR1 activity upon formation of the ETR1-ERS1 heteromeric complex. Alternatively, ERS1 may partially titrate out available RTE1, which promotes ETR1 signaling. CTR1, a Raf-like protein, may directly relay the receptor signal to repress the ethylene response [31,32]. Another possibility is that ERS1 may inhibit CTR1 activity in the presence of ETR1.

Heterosis may occur upon mixture of different genetic backgrounds [27]. Several lines of evidence suggest that the growth recovery by *ers1 *mutations in our study was unlikely to be result of ecotype mixture. The *ers2 *allele is in Ws and the addition of *ers2 *to receptor mutants elevated growth inhibition, rather than causing growth recovery. Additionally, complementing *ers1 *by a Col-0 *ERS1p:ERS1 *transgene in *ers1-2 etr2 ein4 *restored growth inhibition. If the growth recovery had been caused by ecotype mixture, *ERS1p:ERS1 *would not be able to restore growth inhibition. We also showed that the ERS1 protein level was related to the degree of growth inhibition, early leaf senescence, and *CHIB *expression levels in *etr2 ein4 ers2*. These data all favor the argument that ERS1 may promote ethylene responses. We do not exclude the possibility that there may be genetic modifier(s) that can cause some degrees of growth recovery upon ecotype mixture. Nevertheless, our results suggest that the effects of those genetic modifiers on growth recovery are far from sufficient to reverse ethylene growth inhibition to the level of *ers1*.

In the ethylene dose-response assay, both *ers1-2 *and *ers1-3 *displayed a slightly shorter seedling hypocotyl than Ws at low ethylene concentrations, and their seedlings were longer than Ws at high ethylene concentrations. ERS1, like other receptors, is able to repress ethylene responses [11,16,21], although it also inhibits growth, as shown in our study. Conceivably, the effects of ERS1 on growth inhibition can be weakened by ERS1 receptor signaling at low ethylene concentrations; thus, *ers1 *displayed stronger growth inhibition than Ws. At high ethylene concentrations, ERS1 receptor signaling is blocked, while the effects of ERS1 on growth inhibition become stronger due to ERS1 accumulation; thus, *ers1 *displayed weaker growth inhibition than Ws. Notably, *ers1-3 *displayed stronger growth inhibition than *ers1-2 *at 10^-1^μL L^-1 ^ethylene, indicating that growth inhibition was partly reversed by residual ERS1 receptor signaling in *ers1-2*. This result lends support to our hypothesis that in wild type the effects of ERS1 signaling on repressing ethylene responses are stronger than the effects of ERS1 on promoting ethylene responses. Thus, *ers1 *displayed growth inhibition at low ethylene concentrations in Ws.

The five ethylene receptors of Arabidopsis are structurally similar to His-kinase proteins. ERS1 and ERS2 lack the receiver domain, the possible functions of which remain elusive. The inhibitory effects of ERS1 on ethylene receptor signaling are unlikely to be due to its lack of the receiver domain, because ERS2 does not have the same effects as ERS1.

Interestingly, *etr1-7 ers1-3 *displays an extremely strong constitutive ethylene response phenotype (Figure 3C) [11,16,21]. Subfamily I receptors are thought to help subfamily II members activate CTR1, allowing signal output to repress the ethylene response [22]. Another argument is that the subfamily I receptors play more dominant roles than subfamily II receptors in the regulation of ethylene signaling, possibly due to His-kinase activity, the ability to associate with CTR1, or higher expression levels [23,24,31,33]. Phenotypic analyses of (*ERS1*)*etr1 etr2 ein4 ers2 *and *ers1-3 *suggest that effects of ERS1 on regulating ethylene signaling are very minor. Although ETR1 alone represses ethylene response to a great extent, according to the *ers1 etr2 ein4 ers2 *mutant phenotype, the *etr1-7 *mutation does not reciprocally result in the de-repression of the ethylene response. It appears that receptor signal strength is not additive. We thus favor the first scenario, in which subfamily I members may be important for subfamily II signaling. We previously showed that Ag(I) fails to restore seedling hypocotyl growth in *etr1-7 ers1-2 *[11], lending support to the hypothesis that subfamily II signaling is dependent on subfamily I. While the loss of a single subfamily I member does not significantly affect subfamily II signaling, ETR1 and ERS1 are functionally redundant in the regulation of subfamily II receptor signal output.

Emergent function refers to features that are accomplished by multiple genes with similar function, but not by single genes [12]. Unique roles of subfamily I receptors in ethylene signaling may imply one emergent function for ETR1 and ERS1. Positive effects of ERS1 on ethylene responses only occur in the presence of ETR1, implying another emergent function of ETR1 and ERS1. Ethylene receptor signaling may thus be regulated at higher levels involving receptor interactions or collaboration, rather than by receptor number or amount.

## Conclusions

Our work, together with previous studies, shows that growth recovery caused by *ers1 *loss-of-function mutations throughout developmental stages in ethylene receptor mutants is *ETR1 *dependent. *ers2*, which is in the Ws ecotype, did not have the same effect as *ers1 *on growth recovery. Complementing the *ers1-2 *mutation with *ERS1p:ERS1 *restored growth inhibition. ERS1 overexpression resulted in elevation in ethylene response phenotypes, including growth inhibition, early senescence, and elevated *CHIB *expression. These results provide support for the argument that growth recovery by *ers1 *mutations is not due to ecotype mixture. ERS1 plays a role in repressing ethylene responses, and has inhibitory effects on ETR1-specific ethylene signaling. Ethylene receptor signaling may be regulated at multiple levels, including positive and negative collaborations among receptors.

## Methods

### Plant material

In addition to previously described mutants [11,12], we obtained multiple mutants by genetic crosses. *ers1-2 *was crossed with *etr2-3 ein4-4 ers2-3*; *ers1-2 ers2-3 etr2-3*, *etr2-3 ers2-3*, *ers1-2 ein4-4 ers2-3*, *ein4-4 ers2-3*, *ers1-2 etr2-3 ein4-4 *and *ers1-2 etr2-3 ein4-4 ers2-3 *were obtained in generations following F2. *etr1-7 *was genetically crossed with *etr2-3 ein4-4 ers2-3*; *etr1-7 etr2-3 ers2-3*, *etr1-7 ein4-4 ers2-3 *and *etr1-7 ers2-3 *were obtained in generations following F2. *ers1-2 ers2-3 *was a progeny of a cross of *ers1-2 *and *ers2-3*. *ers1-3 *was genetically crossed with *etr2-3 ein4-4 ers2-3*; and *ers1-3 ein4-4 ers2-3 *and *ers1-3 etr2-3 ein4-4 ers2-3 *were obtained. Each of these mutants was confirmed by genotyping and sequencing (data not shown) according to Xie *et. al *[11]. Plant growth conditions were as described [11].

### Seedling triple-response assay

Seedlings were stratified at 4°C for 4 d (96 h) and then transferred to 22°C for 72 h in the dark for germination. The seedling triple response was scored by the measurement of hypocotyl length [13] and at least 20 seedlings (*n *> 20) were scored for each measurement. Ethylene concentrations were measured by gas chromatography (Agilent Technologies, 6890N Network GC System). AVG treatment was followed as described [11,13].

### Gene expression analysis

Total RNA was isolated as described [34] and quantified using a NanoDrop^® ^ND-1000 Spectrophotometer (NanoDrop Technologies, Inc. Welmington, DE, USA). qRT-PCR was performed using a Rotor-Gene 3000 (Corbett Research) and SYBR Premix EX Taq (Takara) to estimate expression of receptor genes, *CHIB*, and *RTE1*. *UBC10 *gene (At5g53300), encoding a ubiquitin-conjugating enzyme, expression was referenced as an internal calibrator. The primer set for *UBI10 *was UBI-F (5'-ATGGAA AATCCCACCTACTAAATT-3') and UBI-R (5'-TTGAACAACTCGTAGCAACTCATC-3'). For gene expression analysis, each RT-PCR was repeated three times from three independent biological materials (*n *= 3 × 3). According to melt curve analysis, the primer sets did not give non-specific PCR amplification across different receptor transcripts (data not shown). The primer set for analysis of *CHIB *expression was F-CHIB (5'-GCCAGACTTCCCATGAAACT-3') and R-CHIB (5'-CAGGGTTGTTGAGTAAGTCA-3'). The primer set for analysis of *RTE1 *expression was RTE1-327F (5'-TCGCTATCTCCAACTCGATAGA-3') and RTE1-629 R (5'-AGACGGTTCAAACAGTTTGCAA-3'). RNA from rosettes was subjected to the analyses for receptor gene expression and the primer sets are shown below.

ETR1 primer set:

F-ETR1 (5'-GCCATCTCCAAGAGGTTTGTGAA-3')

R-ETR1 (5'-CCGTTCTCATCCATGACAAGA-3') ERS1 primer set:

F-ERS1 (5'-CTGATTCTGTCTGCAGA-3')

R-ERS1 (5'-TGTGTGAATTCCACACCCTGTG-3') ETR2 primer set:

F-ETR2 (5'-GAAAGTGGTGCAGTTGATTCAT-3')

R-ETR (5'-CGAATCGTTGGTGTCTACCA-3') ERS2 primer set:

F-ERS2 (5'-GCCAAAACATTGTAAAGTATATGCA-3')

R-ERS2 (5'-CTTCCTGACGTCAATGATCAGT-3') EIN4 primer set:

F-EIN4 (5'-ACTTGCACAGATGATGCA-3')

R-EIN4 (5'-GACATCATCATCGTCTGCTA-3')

Receptor regions subjected to qRT-PCR analysis were sequenced. No polymorphisms were found at the priming regions in Ws and Col-0 (data not shown). The transcript copy number of each receptor gene in wild type was estimated by qRT-PCR and the total receptor gene transcript copy number (Cwt) was obtained by adding the transcript copy number of each receptor gene. The total transcript copy number of the remaining wild-type receptor genes in a receptor mutant (Cmt) was estimated using the same method. The relative total receptor gene expression (Cmt/Cwt) was estimated by dividing the receptor gene transcript copy number in a receptor mutant (Cmt) by the total receptor gene transcript copy number in wild type (Cwt). The relative total receptor gene expression in wild type was referred to as 1 (Cwt/Cwt = 1).

### Statistics

Student's *t *test was used for comparisons between two measurements. LSD-t test (least significant difference *t *test) was used for multiple paired comparisons. The *F *test was used for comparisons among measurements greater than two means. Unless specified, the error rate α = 0.01 was used in all comparisons. The *P *value indicates the probability of a numerically larger value of *t *in Student's *t *test or LSD-t test. In the ethylene dose-response assay, the LSD-t test was used to compare means of the measurement at each ethylene concentration.

### Cloning the *ERS1 *transgene

A genomic *ERS1 *clone was released from a BAC clone (T20B5) by *Spe *I and *Kpn *I. The resulting *ERS1 *fragment was cloned into pBJ36, in which an *OCS *terminator follows the *ERS1 *fragment. *ERS1-OCS *was next released by *Not *I and cloned to a binary vector pMLBart for *Agrobacterium*-mediated transformation. To clone *ERS1 *into a *35S *promoter-containing binary vector, an *ERS1 *fragment (623-1173 bp) was released by *Hind *III and *Sph *I from the BAC clone T20B5. The resulting fragment was ligated to the *Sph *I site on a cDNA fragment (1173-stop). The full-length *ERS1 *was released by *Hind *III and *Xba *I and sub-cloned into the binary vector pHB for transformation.

### Antibodies and immunoassay

The *ERS1 *cDNA fragment encoding ERS1(158-407) was cloned into the expression vector *pET28b*. The antibody against ERS1(158-407), ERS1-Ab, was prepared by the Antibody Research Center of SIBS (Shanghai Institutes for Biological Sciences). For immunoassay, ERS1 protein was detected by ERS1-Ab; the goat anti-rabbit IgG conjugated with peroxidase (ImmuClub; part no. SA0040), and the ECL reagents (Amersham) detected ERS1-Ab. Chemiluminance was captured on Kodak film (XBT-1) or by a cold CCD (Versa Arra^® ^Systemy, Princeton Instruments, Roper Scientific, Inc.). After exposure, the immunoblot was stained with Coomassie Blue.

## Authors' contributions

C-KW: designed the research, analyzed data, and wrote manuscript. QL: obtained receptor mutants by genetic crosses, performed experiments, and analyzed data. CX: performed qRT-PCR and analyzed gene expression. All authors read and approved the final manuscript.
